# Psychometric Properties of the Iranian Version of Modified RHAQWRA Questionnaire

**Published:** 2015-11

**Authors:** Roghaieh Rahmani Bilandi, Farideh Khalajabadi Farahamni, Fazlollah Ahmadi, Anoshirvan Kazemnejad, Reza Mohamadi, Mostafa Amiri

**Affiliations:** 1 Department of Reproductive Health, Faculty of Medical Sciences, Tarbiat Modares University, Tehran, Iran; 2 National Population Studies and Comprehensive Management Institute, Tehran, Iran; 3 Department of Nursing, Faculty of Medical Sciences, Tarbiat Modares University, Tehran, Iran; 4 Department of Biostatistics, Faculty of Medical Sciences, Tarbiat Modares University, Tehran, Iran; 5 Crisis Manangment Working Group, Tehran, Iran; 6 Department of Basic Sciences, Faculty of Allied Medicine, Gonabad University of Medical Sciences, Gonabad, Iran

**Keywords:** Women, Reproductive Health, Disaster, Psychometric Properties, Questionnaire

## Abstract

**Objective:** This study aimed to examin the psychometric characteristics of the Persian version of Reproductive Health Assessment Questionnaire for Women of Reproductive Age (RHAQWRA).

**Materials and methods:** This study was a cross sectional study. We selected 150 women aged 18- 45 years based on convenience sampling method who lived in the Quake-hit areas in eastern part of Iran in 2013. Some previously recognized and established instructions were undertaken in translating and adapting the RHAQWRA questionnaire culturally. We sought help and feedback from teenagers and experts in the fields of midwifery, sociology, and epidemiology to establish the face and content validity of the questionnaire. To examine the test-retest reliability indices of all the items, we used Kappa and McNemar tests.

**Results:** The Content Validity Index (CVI) and Content Validity Ratio (CVR) scores for RHAQWRA were above 0.70. Results from Kappa and McNemar tests indicated a high degree of test-retest reliability. In order to evaluate the construct validity, known- group comparison (women with and without unwanted pregnancy) was used. The internal consistency reliability indexes for the subscales obtained through Cronbach’s alpha were between 0.68 and 0.87.

**Conclusion:** The findings suggest that the Iranian version of RHAQWRA is a valid and reliable questionnaire which can be used for measuring reproductive health needs during disasters.

## Introduction

The incident of the natural and man-made disasters in the world is growing (1). In Iran, during the recent years, many earthquakes have occurred and led to the death of 30,000 people (2). Also, some people have been injured (3), and there has been more than a billion dollars in property loss (4). This demonstrates the importance of health emergency preparedness, prevention, and response programs (1). 

During a disaster, women, for various reasons, are at much greater risk of death than men (5). The female gender is a risk factor which forms the basis for developing post-disaster psychopathologies (6). So, there exists some evidence that disaster influences the mother’s mental health and leads to some perinatal health outcomes and men’s health particularly among highly-exposed women (7).

Earthquakes increase stress and have been associated with an increased incidence rates of low birth weight, preterm labour, birth defects, lower Apgar scores (8) and in the baby’s subsequent development (9). Gender-based violence has also increased in disasters (10). Therefore, reproductive health is important in disasters. We focused on RHAQWRA (Reproductive Health Assessment Questionnaire Women of Reproductive Age), the only one specific scale existing to measure the reproductive health needs of women in disasters. RHAQWRA designed by Jennifer Horney contains items that measure 5 subscales: Pregnant Women, Post-Partum Women & Their Infants, Family Stressors, Risk Behaviors and Needs, and Violence (11).

The RHAQWRA was originally designed in English for use in the USA. We decided to translate the RHAQWRA from English to Persian. As such, using the exiting questionnaire in different countries required cultural adaption. In this study, to ensure on the cultural appropriateness of the questionnaire for our sample, we assessed its reliability, validity and its cultural relevance and assumed that it would provide us with appropriate and essential data on topics related to the management disaster which might be used in future studies in Iran.

## Materials and methods

***The RHAQWRA questionnaire***

The RHAQWRA questionnaire is a specific and self-report instrument that focuses on women’ needs in disasters. It consists of 52 items and almost all of the items are rated on a yes/no basis. 

***1. Translation and cultural adaptation***

Having obtained permission from Jennifer Horney, the developer of the RHAQWRA, we conducted a forward-backward procedure to translate the English version of the RHAQWRA into Persian. Translation is the most common method of preparing questionnaires for cross-cultural application (12).

We did the translation and cross-cultural adaptation research according to Beaton and et al. (13) that Translation of a scale is important (14). 

Each stage in the process of translation is described in detail in what follows:

Stage1: Item content, response options, and instructions were all translated in this stage by two translators (12). One of the translators was aware of the concepts in reproductive health. The other translator had no background information in the field.

Stage II: The two translators and one of the authors compared the translations and produced a final version of the questionnaire. 

Stage III: Two English teachers back translated the Persian questionnaire into English. Through this stage, it is checked whether the content of translated version exactly corresponds with that of the original version of the questionnaire (13).

Stage IV: The researchers, one specialist in psychometrics, two midwives, two nurses, and one epidemiologist reviewed all the translations and the differences were discussed by these experts who formed a committee. To achieve a kind of cross-cultural equivalence, the committee framework is of high importance (12).

Stage VI: Then, the Persian RHAQWRA was adjusted. In the Persian questionnaire, there was one modification in item "Community health clinic and Public health clinic" which was changed to "health home and health center" to adapt to the Iranian culture.

In the final stage of the adaptation process, all forms and reports was submitted to the questionnaire developer and the committee responsible for tracking the translated version of this instrument (12).

***2. Design and data collection***

This was a cross-sctioneal study of women living in the earthquake-stricken area of Zohan city in the eastern Iran. The sample were 150 women who based on convenience sampling method and entered the study if they were 18-45 years of age, had no problems in speaking or listening, and lived in the earthquake area in 2013. The aim of the study was explained to women and written consent was received.

*2.1. Face Validity*

Face validity assessment refers to the fact that whether a questionnaire items possess relevance, reasonability, and clarity (15). and detects any inappropriate item (16).

In our study, we investigated face validity qualitatively and quantitatively. With regard to the qualitative stage, the questionnaire was assessed by ten women; and the data collected from face to face interviews were examined. In the quantitative stage, the 55 items in the questionnaire were scored based upon a five-point Likert scale type. During this stage, to show the percentage of the women who recognized an item important or quite important, the impact score, i. e., frequency × importance, was calculated. The impact score ranged from 1.2 to 5. The items were acceptable if they had an impact score equal to or greater than 1.5.

*2.2. Content validity*

Content validity refers to the extent to which constituents of testing instrument enjoy relevance and representativeness of the intended construct for a specific testing aim (17). Ten specialists in the fields of midwifery, medicine, nursing, and disaster managements were asked to comment independently on including or excluding an item in order to calculate the content validity ratio (CVR).

The CVR for the total scale was computed by the formula after the participants responded to each of the items based on a spectrum with three aspects of item is essential, is useful but not essential, and is not necessary. According to Lawshe’s table, an acceptable CVR value for 10 specialists is 0.62 (18). 

The CVR is an item statistic which is helpful in rejecting or retaining specific items. It is calculated for the entire test after the items have been identified and decided upon to be included in the final form of the test. It is merely the mean of the CVR values for all the items retained. Hence, it is of importance to emphasize that the CVI should not be confused with a the correlation coefficients (18).

Consequently, CVI assesses the relevance and simplicity of an item to the content represented in the questionnaire (19).

Then, the CVI was computed based on the formula (20). The criteria used regarding the quantitative values of CVI were as follows: if the values were below 0.70: they were considered as unacceptable; if they were 0.70-0.78, they needed revision and correction; and if they were equal or above 0.79, they were assumed to be suitable.

Finally, items (n = 21) with inappropriate scores in all three measurements of impact factor score, CVI, and CVR for validity questionnaire were revised and modified.

*2.3. Construct validity:*

Construct validity shows the degree to which a variable actually reflects the theoretical meanings of a concept (15). In this regard, we used known-groups comparisons to find out whether the questionnaire could distinguish between groups of women with various pregnancy and reproductive health conditions. If we expected differences, we made use of comparison between subgroups (21). The comparison between the subgroup consisted of Pregnant Women, Post-Partum Women & Their Infants, Family Stressors, Risk Behaviors and Needs, and Violence. Pregnancy state (unwanted pregnancy) is an important issue in the interpretation of differences in RHAQWRA. Thus, the subscales in the two groups of women with regard to the wanted and unwanted pregnancies were compared. Group differences were assessed using Mann-Whitney U-tests. 

***3. Reliability***

The reliability of the questionnaire was assessed by Cronbach’s alpha coefficient. Cronbach (1951) developed a formula for estimating the internal consistency of an instrument in which the items are not scored dichotomously (e.g. Yes/No, Right/Wrong, True/False, Agree/Disagree, etc.) (15). 

However, the Values equal or greater than 0.70 were considered satisfactory in the study (22). Also, to estimate the reliability indexes of the RHAQWRA and its subscales, test-retest and internal consistency were utilized. To do so, we selected fifty women with the age range of 18-45 years through random sampling.

The women were instructed to check each item. They filled out the RHAQWRA twice during a period of two weeks. The test-retest reliability and the internal consistency indices were computed by using Kappa tests and Cronbach’s alpha coefficient, respectively. 

The reference values for the Kappa coefficients were based (23) who considers values ≤ 0.40, 0.41-0.75, and > 0.75, as poor, moderate to good, and excellent, respectively. SPSS V.18 was used to analyze the data. The p values < 0.05 were considered as significant. 

***4. Ethics***

The ethical approval for this study was obtained from the Ethics Committee of the Tarbiat Modares University (Code of ethics: 92-11-20); and permission for translation, modification, and use of the RHAQWRA was obtained from Professor Jennifer Horney.

## Results

In all, 150 women living in the earthquake-stricken area completed the questionnaire. Socio-demographic data of the women are presented in table 1.

**Table 1:** Socio-demographic information in Iranian women

**Variable**		
Age in years, mean (SD)	35.9 (7.36)	
Education, n (%)		
Guidance school or less	122 (78%)	
Completed high school	16 (10%)	
College	19 (12%)	
Marital status		
Married	129 (82.2%)	
Divorced	2 (1.3%)	
Widowed	18 (11.5%)	
Separated	2 (1.3%)	
Never married	6 (3.8%)	
Home damage		
No damage	5 (3.2%)	
Minor	7 (4.4%)	
Major	26 (16.4%)	
Destroyed	117 (74.5%)	
Some people in residence		
Babies (<1 year)	15	
Children and teenagers (1-17 years)	126	
Adults (18-64 years)	107	
Older adults (≥65 years)	26	
Total number of people health insurance, n (%)		
Social	39 (24.8%)	
Medical service	103 (65.6%)	
Other	13 (7.8%)	

***Face validity***

Impact score was calculated to examine quantitative face validity. The results revealed that almost all the items had an impact score ≥ 1.5. These items were important in the target group but nationality (being Iranian), race, and alcohol consumption items had the Impact sore < 1.5. In the qualitative stage, some minor wording changes were done according to women’s suggestion.

***Content validity***

The CVR for most of the items was more than that of Lawshe’s Scale so the items proved acceptable. For some items related to nationality, race, neonatal weight, neonatal age, alcoholic consumption, and sex, CVI was < 0.80. After revising these items, CVI for neonatal weight, neonatal age, and sex were ≥ 0.80. However, nationality, race, and alcoholic consumption items had inappropriate score.

***Construct validity***

The results related to the methods of known-groups comparison indicated a relation between sub-scores to wanted and unwanted pregnancies. The results of the answers for each item in the two groups, wanted and unwanted pregnancies, were: pregnancy (p ≤ 0.03), post-partum (p ≤ 0.04), family planning (p ≤ 0.005), need (p ≤ 0.018), and violence (p ≤ 0.002). Therefore, the results were acceptable.

***Reliability***

The results of the analyses are provided in table 2. The items: Pregnant Women, Post- Partum Women & Their Infants, Family Stressors, Risk Behaviors, and Needs had Cronbach’s Alpha ≥ 0.70. Only one item, violence, had Cronbach’s α < 0.70. According to the Gordis result, the Kappa coefficients of the RHAQWRA items which were between 0.40 and 0.75 or above 0.75 indicated a suitable agreement of this instrument (22).

The distribution of the answers for each item and scale are shown in table 2 (the first column). The majority of missing data were from items related to violence issues, with 11% of the answers to sexual functioning missing. The answers to “the sex with more than one person” item had 7% missing. Surprisingly, only 64 (27.1%) women answered the item risk of getting HIV or any sexually transmitted infection. There was a much lower response rate to the equivalent questions among women. It seems that there is a need to educate these women.

## Discussion

The assessment of reproductive health needs in women in the disaster is necessary (24). This study is the first to describe and examine the psychometric characteristics of the RHAQWRA in Iran. 

The study investigated the relation between the needs of reproductive health and wanted or unwanted pregnancies. And the needs were almost related to medicine; and its prevalence was 64%. But in the study conducted by Simpson, the most common social need was the counseling services and information for the person and their family; and the need for the medical services and the physician’ presence was 8.9% (25). Also, in our study, walking through the debris was a stressor, similar to what was reported by Simpson (25).

**Table 2:** Distribution of Test-retest reliability scores and Cronbach’s alpha of RHAQWRA in Iranian women

Item	McNemar					
	p	Kappa	CVI	CVR	IF	Cronbach alpha
**Pregnant Women**						
** Currently pregnant**	**0.89**	**0.79**	**1**	**1**	**4.7**	**0.9**
** How far along pregnancy**	**0.96**	**1**	**4**			
** First week to visit for prenatal care**	**0.9**	**1**	**3.37**			
** Currently to see someone for prenatal care**	**1**	**0.87**	**1**	**1**	**4.5**	
** Things kept you from getting prenatal care**	**0.94**	**0.8**	**4.2**			
** Not enough money**	**1**	**0.83**				
** Transportation**	**1**	**0.82**				
** Many other things**	**1**	**0.83**				
** Has not time off**	**1**	**0.93**				
** No one to take care**	**1**	**0.89**				
** Difficulties for prenatal care**	**1**	**0.86**	**1**	**1**	**2.88**	
** Health problems**			**0.96**	**1**	**2.49**	
** Asthma**	**1**	**0.95**				
** Vomiting**	**1**	**0.82**				
** Hypertension**	**1**	**0.95**				
**Post Partum Women & Their Infants**						
** Baby six months old**	**0.87**	**0.83**	**1**	**1**	**3.8**	**0.79**
** Pregnant when disaster**	**1**	**0.84**		**0.80**	**0.93**	**3.6**
** Baby weighs at birth**	**0.8**	**0.80**	**2.1**			
** Preterm labor**	**1**	**0.92**	**0.94**	**0.8**	**2.1**	
** Postpartum checkup**	**1**	**0.95**	**1**	**1**	**3.55**	
** More difficult to get postpartum checkup**	**0.79**	**0.77**	**0.96**	**1**	**3.8**	
** Reason for not getting a postpartum checkup**	**0.8**	**.80**	**3.12**			
** Not enough money**	**1**	**0.92**				
** No transportation**	**0.78**	**0.65**				
** Baby checkup with a doctor**	**1**	**0.91**	**1**	**1**	**4.6**	
** More difficult to baby checkup**	**0.77**	**0.57**	**1**	**1**		**3.6**
** Things keep baby from having a checkup**	**0.8**	**0.8**	**3.12**			
** Not enough money**	**1**	**0.92**				
** No transportation**	**0.82**	**0.76**				
** Baby was sick**	**0.87**	**0.76**				
** Have nowhere for care**	**0.66**	**0.54**				
** Breastfeeding**	**0.59**	**0.57**	**0.8**	**0.8**	**3**	
** Health**	**1**	**1**	**0.86**	**1**	**4.8**	
** Have difficulty to get formula**	**1**	**1**	**1**	**1**	**3.8**	
** Have difficulty getting clean water to clean bottles**	**0.6**	**0.59**	**4. 6**			
**Family Planning**						
** Tubes tied or vasectomy**	**0.76**	**0.69**	**0.86**	**0.75**	**0.8**	**4.6**
** Using ** ***any*** ** birth control method**	**1**	**0.95**	**1**	**0.8**	**4.5**	
** Difficult to get your birth control method**	**0.87**	**0.78**	**1**	**1**	**3.82**	
** Anything** ** now to keep from getting pregnant**	**1**	**0.95**	**1**	**1**	**4.2**	
** Reasons for not doing ** ***anything *** **to keep from getting pregnant now**	**0.86**	**0.8**	**3.8**			
** Not have sex**	**0.98**	**0.87**				
** Want pregnant**	**1**	**0.98**				
** Pregnant now**	**0.98**	**0.87**				
** Do not want to use**	**0.65**	**0.49**				
** My husband do not want to use**	**0.87**	**0.69**				
** Kind of birth control method**	**1**	**0.78**	**4.5**			
** Pill**	**1**	**1**				
** Condoms**	**0.87**	**0.78**				
** Depo-Provera®**	**1**	**0.96**				
** IUD**	**1**	**0.98**				
** Where obtain birth control**	**0.86**	**0.8**	**3.8**			
**Family Stressors, Risk Behaviors, and Needs**						
** Health problems**	**0.8**	**1**	**3.9**	**0.83**		
** High blood sugar (diabetes)**	**1**	**0.97**				
** Asthma**	**0.44**	**0.39**				
** Kidney or bladder (urinary tract) infection**	**0.79**	**0.46**				
** High blood pressure, hypertension**	**1**	**0.95**				
** Heart problems**	**1**	**0.95**				
** Experience of the disaster**	**1**	**1**	**1**	**1**	**4.2**	
** Life in danger**	**0.9**	**0.88**	**0.59**	**0.55**	**4.5**	
** Illness or injury**	**0.72**	**0.66**	**0.84**	**0.80**	**4.6**	
** Illness or injury in the household**	**0.64**	**0.60**	**0.8**	**0.8**	**4**	
** Walking in the debris**	**1**	**0.91**	**0.8**	**1**	**4.6**	
** Without electricity**	**1**	**0.99**	**1**	**1**	**4.19**	
** Someone died**	**0.8**	**0.74**	**1**	**1**	**3.5**	
** Saw someone die**	**1**	**0.94**	**0.9**	**1**	**3.7**	
** Living alone**	**1**	**0.91**	**0.93**	**1**	**4.8**	
** Lost personal belongings**	**0.83**	**0.80**	**1**	**1**	**4.7**	
** Separated from loved ones**	**0.9**	**0.86**	**0.8**	**0.8**	**4.4**	
** Had difficulty for getting government services**	**0.47**	**0.42**	**1**	**1**	**3.82**	
** Lost job**	**0.81**	**0.76**	**0.93**	**1**	**3.7**	
** Lost job when wanted**	**1**	**0.91**	**0.8**	**1**	**2.94**	
** Arguing with husband**	**0.75**	**0.68**	**0.86**	**0.80**	**4.7**	
** Could not pay bill**	**0.81**	**0.76**	**1**	**1**	**3.7**	
** Physical fight**	**0.87**	**0.78**	**0.86**	**0.8**	**1.86**	
** Before the disaster, the number of people in your household**	**0.9**	**0.8**	**1.65**			
**Since the disaster, the number of people in your household**	**1**	**3.8**	**1.92**			
** Before the disaster, station food**	**1**	**0.99**	**0.94**	**0.8**	**4.1**	
** Since the disaster, station food**	**1**	**0.88**	**0.8**	**0.94**	**3.6**	
** Felt unsafe in the place before the disaster**	**0.9**	**1**	**3.37**			
** Felt unsafe in the place since the disaster**	**0.9**	**1**	**4.4**			
** Used tobacco**	**0.99**	**0.92**	**0.9**	**1**	**4.7**	
** Since the disaster, how many alcoholic drinks have you had in an average week**	**0.64**	**0.60**	**1.2**			
** Sex with more than one person**	**0.69**	**0.92**	**0.8**	**0.90**	**3.7**	
** Risk of getting HIV or STI**	**0.87**	**0.77**	**0.90**	**1**	**4.6**	
** Reasons for risk of getting HIV or STI**	**0.08**	**0.08**	**2.3**			
** Currently need**						
** Housing**	**0.73**	**0.70**	**1**	**1**	**4.8**	
** Clothes**	**1**	**0.96**	**0.96**	**1**	**4.1**	
** B) Money to buy food**	**1**	**0.92**	**1**	**1**	**2.8**	
** C) School or vocational training**	**0.81**	**0.73**	**0.93**	**1**	**1.86**	
** D) Transportation**	**0.59**	**0.63**	**1**	**1**	**2.85**	
** E) Dental services**	**0.56**	**0.69**	**1**	**1**	**4.1**	
** F) Medical services**	**0.52**	**0.51**	**1**	**1**	**4.1**	
** G) Help to quit smoking**	**0.76**	**0.68**	**0.93**	**0.80**	**2.5**	
** Need to Help with alcohol or drug problem I**	**0.81**	**0.72**	**1**	**0.80**	**2.8**	
** Help to reduce violence in your home**	**0.59**	**0.63**	**0.8**	**0.93**	**4.2**	
** J) Counseling for family problem**	**1**	**0.96**	**1**	**0.8**	**3.6**	
** Method of getting health information**	**0.85**	**1**	**1**	**3.63**		
** Violence**						
** Partner pushed, physically hurt**	**0.88**	**0.79**	**0.86**	**1**	**4.9**	**0.64**
** Mistreated you physically**	**0.80**	**0.76**	**0.93**	**0.80**	**2.34**	
** Force you to take part in touching**	**1**	**0.90**	**0.86**	**0.80**	**3.11**	
** Believe that violence affected health**	**0.90**	**0.89**	**0.93**	**1**	**3.91**	
** Medical care provider for treatment**	**0.34**	**0.60**	**0.93**	**1**	**3.13**	

In translation and cultural adaptation of RHAQWRA, we were not faced with serious problems. As such, it was not necessary to make major changes to its original form. Slight changes were done in the questionnaire, that is, some parenthetical explanations were given for some items. So, we found a high correlation between the English and Persian forms of RHAQWRA, and its validity and reliability indices were satisfactory.

The test-retest reliability and Kappa values were acceptable for all items, and all items had a Cronbach's α > 0.70. Only violence item had a Cronbach's α < 0.70 (0.64). This may have been hidden with gender and culture problem because these items are naturally sensitive for the Iranian community.

This level of test-retest reliability is comparable to that of a previous study (8).

In this study, for face validity, the women found the questionnaire items interesting. The findings indicated that 70% of women believed that nationality was not a necessary item as almost Iranian women were interviewed. 

Regarding content validity, our study revealed that the items which had a CVI > 0.80 were accepted. Nationality and alcohol consumption had a CVI < 0.60.

In summary, the present study confirmed validity and reliability of the Iranian version of RHAQWRA. However, it should be noted that it has certain limitations. The assessment of reproductive health needs of women living in Iran may limit generalization of the finding to all Iranian women. This questionnaire has not been used in Iran so far, but some studies in other countries have established the reproductive health needs of women in post-disaster conditions (26).

Disaster has been associated with a decrease in access to healthcare and medications, increased maternal risk factors, and poor birth outcomes (27) . Reproductive health is both a significant public health issue including emergencies and a human right one (28). Assessment reproductive health needs women is necessary (29) and this is an innovative way (30).

## Conclusion

Assessing women’s needs by RHAQWRA promoted the women’s health.and can be used by public health researchers and practitioners to assess and prioritize the reproductive health needs in disasters; and it can be considered as one source of information from which the reproductive health promotion programs and policies can benefit.
